# Disruption of the mitochondrial alternative oxidase (AOX) and uncoupling protein (UCP) alters rates of foliar nitrate and carbon assimilation in *Arabidopsis thaliana*

**DOI:** 10.1093/jxb/eru158

**Published:** 2014-05-05

**Authors:** Anthony Gandin, Mykhaylo Denysyuk, Asaph B. Cousins

**Affiliations:** School of Biological Sciences, Molecular Plant Sciences, Washington State University, Pullman, WA 99164-4236, USA

**Keywords:** Alternative oxidase, ammonium, energy balancing, nitrate assimilation, reductant, uncoupling protein.

## Abstract

Under high light, the rates of photosynthetic CO_2_ assimilation can be influenced by reductant consumed by both foliar nitrate assimilation and mitochondrial alternative electron transport (mAET). Additionally, nitrate assimilation is dependent on reductant and carbon skeletons generated from both the chloroplast and mitochondria. However, it remains unclear how nitrate assimilation and mAET coordinate and contribute to photosynthesis. Here, hydroponically grown *Arabidopsis thaliana* T-DNA insertional mutants for alternative oxidase (AOX1A) and uncoupling protein (UCP1) fed either NO_3_^–^ or NH_4_^+^ were used to determine (i) the response of NO_3_^–^ uptake and assimilation to the disruption of mAET, and (ii) the interaction of N source (NO_3_^–^ versus NH_4_^+^) and mAET on photosynthetic CO_2_ assimilation and electron transport. The results showed that foliar NO_3_^–^ assimilation was enhanced in both *aox1a* and *ucp1* compared with the wild-type, suggesting that foliar NO_3_^–^ assimilation is probably driven by a decreased capacity of mAET and an increase in reductant within the cytosol. Wild-type plants had also higher rates of net CO_2_ assimilation (*A*_net_) and quantum yield of PSII (ϕ_PSII_) under NO_3_^–^ feeding compared with NH_4_^+^ feeding. Additionally, under NO_3_^–^ feeding, *A*_net_ and ϕ_PSII_ were decreased in *aox1a* and *ucp1* compared with the wild type; however, under NH_4_^+^ they were not significantly different between genotypes. This indicates that NO_3_^–^ assimilation and mAET are both important to maintain optimal rates of photosynthesis, probably in regulating reductant accumulation and over-reduction of the chloroplastic electron transport chain. These results highlight the importance of mAET in partitioning energy between foliar nitrogen and carbon assimilation.

## Summary

We demonstrated that mitochondrial respiration contributes to energy balancing between nitrogen and carbon metabolism by competing for reductant with *de novo* NO_3_^–^ assimilation and avoiding chloroplastic excess reductant.

## Introduction

Nitrogen (N) availability is a major determinant of plant growth and productivity (Reich *et al.*, 1997, 2006a). However, N fertilizers also represent a large economical cost and source of ground water pollution in agriculture systems. In higher plants, nitrate (NO_3_^–^) and ammonium (NH_4_^+^) are the two primary forms of assimilated inorganic N. The latter is typically assimilated directly into amino acids through the glutamine synthetase–glutamate synthase complex, whereas the former is first reduced to nitrite by cytosolic nitrate reductase and then to NH_4_^+^ by plastidic nitrite reductase (Epstein and Bloom, 2005). The assimilation of NO_3_^–^ has a higher energetic requirement compared with NH_4_^+^ assimilation (Noctor and Foyer, 1998; Scheurwater *et al.*, 1999; Escobar *et al.*, 2006) and in the leaves the cytosolic reduction of nitrate to nitrite may consume reductant exported from either the chloroplast or the mitochondria (Foyer *et al.*, 2011). On the other hand, nitrite reduction and NH_4_^+^ assimilation occur in the chloroplast stroma and consume reduced ferredoxin. Therefore, the complexity and compartmentalization of these pathways necessitates balancing photosynthetic energy supply and demand to optimize rates of NO_3_^–^ and CO_2_ assimilation.

The availability of N is known to affect rates of photosynthesis and respiration (Reich *et al.*, 2006b; Atkinson *et al.*, 2007), and there is a strong correlation between leaf N content and rates of respiration (Terashima and Evans, 1988; Makino and Osmond, 1991; Byrd *et al.*, 1992; Lusk and Reich, 2000). For example, the tricarboxylic acid (TCA) cycle enzymes fumarase, NAD-dependent isocitrate dehydrogenase (Makino and Osmond, 1991), and NAD-dependent malic enzyme (Noguchi and Terashima, 2006) are up-regulated under low N. Additionally, the expression and activity of several glycolytic and TCA cycle enzymes were differentially influenced following NH_4_^+^ or NO_3_^–^ feeding (Larsen *et al.*, 1981; Scheible *et al.*, 1997; Lancien *et al.*, 1999; Stitt, 1999). These changes in respiration are linked to the supply of carbon skeletons (e.g. 2-oxoglutarate, isocitrate, and citrate) from the TCA cycle to maintain optimum N assimilation and amino acid biosynthesis (Larsen *et al.*, 1981; Scheible *et al.*, 1997; Lancien *et al.*, 1999). Furthermore, the N source (NO_3_^–^ versus NH_4_^+^) has been shown to change gene expression of the mitochondrial electron transport chain, particularly the alternative oxidase (AOX), type II NAD(P)H dehydrogenases, and uncoupling proteins (UCPs) of the mitochondrial alternative electron transport (mAET) (Escobar *et al.*, 2006; Patterson *et al.*, 2010). Additionally, the capacity and protein amount of AOX, a major component of mAET, increase under low NO_3_^–^ conditions (Sieger *et al.*, 2005; Watanabe *et al.*, 2010; Hachiya and Noguchi, 2011). The mAET bypasses one or more of the multiprotein complexes of the ‘classic’ electron transport chain, minimizing proton pumping across the inner membrane (Vanlerberghe and McIntosh, 1997; Rasmusson *et al.*, 2004). Consequently, mAET oxidizes NAD(P)H uncoupled from ATP production and has been proposed to dissipate excess reductant (Raghavendra and Padmasree, 2003). For example, mAET plays an important role in the response to several environmental constraints such as cold (Armstrong *et al.*, 2008; Watanabe *et al.*, 2008), elevated CO_2_ (Gandin *et al.*, 2012), drought (Bartoli *et al.*, 2005; Giraud *et al.*, 2008), phosphate limitation (GonzàLez-Meler *et al*., 2001; Sieger *et al.*, 2005; Escobar *et al.*, 2006), high light stress (Ribas-Carbo *et al.*, 2000; Sweetlove *et al.*, 2006; Yoshida and Noguchi, 2009), and other reactive oxygen species-inducing stress conditions (Maxwell *et al.*, 1999). Additionally, it has been suggested that the capacity of mAET responds to changes in NO_3_^–^ assimilation (Dutilleul *et al.*, 2005; Escobar *et al.*, 2006).

Reductant availability within the leaf cytoplasm probably often limits rates of *de novo* NO_3_^–^ assimilation. This is in part due to the low cytosolic NADH availability (0.3–0.7 μM), which is far below the 7 μM *K*_m_ of nitrate reductase for NADH (Kaiser *et al.*, 2000; Heineke *et al.*, 2001). Therefore, conditions such as high light or perhaps high rates of photorespiration that increase cytosolic NADH concentrations have been suggested to increase rates of foliar NO_3_^–^ assimilation (Bloom *et al.*, 2002; Searles and Bloom, 2003; Rachmilevitch *et al.*, 2004; Guo *et al.*, 2007). Alternatively, NO_3_^–^ assimilation decreases under conditions that restrict the export of reductant via the malate shuttle from the chloroplast. It has also been reported in the literature that changes in mitochondrial electron transport, particularly the alternative non-phosphorylating pathways, could influence the *de novo* assimilation of N (Watanabe *et al.*, 2008). However, it remains unclear how the alternative non-phosphorylating pathways of the mitochondrial inner membrane influence foliar NO_3_^–^ assimilation.

Additionally, it has been shown that both the alternative non-phosphorylating pathways of the mitochondrial inner membrane and N assimilation oxidize excess reductant produced by photosynthesis, photorespiratory glycine oxidation, and the TCA cycle (Sweetlove *et al.*, 2006; Gandin *et al.*, 2012). Therefore, the energy partitioning in the cell is probably balanced in part by both mitochondrial electron transport and NO_3_^–^ assimilation (Hachiya and Noguchi, 2011), avoiding chloroplast over-reduction and maintaining optimal rates of photosynthesis. The role of the mAET and NO_3_^–^ assimilation in energy consumption and dissipation is well accepted; however, their respective contribution and coordination remain unclear. Therefore, the aim of this research is to test the hypothesis that changes in mAET and NO_3_^–^ assimilation influence energy partitioning between N and carbon metabolism. To test this hypothesis, this study (i) investigated the influence of changes in mAET capacity on *de novo* NO_3_^–^ assimilation and (ii) determined the response of photosynthetic CO_2_ assimilation and electron transport to changes in N source (NO_3_^–^ versus NH_4_^+^) in plants with disrupted mAET [wild type (WT) versus *aox1a* and *ucp1*].

## Materials and methods

### Plant material and growth conditions

WT and T-DNA insertion lines for AOX1a (SALK_084897) and UCP1 (SAIL_536G01) plants of *Arabidopsis thaliana* (Sweetlove *et al.*, 2006; Giraud *et al.*, 2008; Gandin *et al.*, 2012) were grown hydroponically in a controlled environment growth chamber (Biochambers GC-16, Winnipeg, Manitoba, Canada) at a photosynthetic photon flux density (PPFD) of 160 μmol quanta m^–2^ s^–1^ at plant height, relative humidity of 50%, and air temperature of 23 °C and 18 °C during the day and night, respectively, with a 10h day. The SAIL_563G01 line was obtained from the TAIR collection and homozygous lines were screened by PCR of genomic DNA using the GACGAAGATGTGAAGTAGACC/TAGCATCTGAATTTCATAACCAATCTCGATACAC and GACGAAGATGTGAAGTAGACC/TCAGTTTCTTTTGGACG CATCG primer pairs. Homozygous lines were selfed and screened again by PCR using the same primer pair. Seeds were germinated on Rockwool cylinders (GroDan Cubes, Rockwool BV, Roemond, The Netherlands) for 7 d. Subsequently, seedlings were transferred to 14 litre containers filled with aerated nutrient solution containing 0.2mM NH_4_Cl, 0.2mM KNO_3_, 1.25mM CaSO_4_, 0.75mM MgSO_4_, 0.5mM KH_2_PO_4_, 0.04g l^−1^ FeDPTA, and micronutrients (Gibeaut *et al.*, 1997). Nutrient solution was replaced every 2 d.

### Growth parameters, chlorophyll contents, and Rubisco activity

Total leaf area, leaf number, and rosette size were measured from digital pictures of whole plants using Image J software (NIH, Bethesda, MD, USA). Rosette size was calculated according to Feret’s diameter principle using ImageJ software (version 1.37, NIH, USA). Additionally, shoot and root were weighed separately (fresh weight), then dried for 96h at 65 °C and weighed again (dry weight). Leaf mass per area (LMA) was estimated as the ratio of dry weight to leaf area. Chlorophyll content was quantified according to Ritchie (2006). Rubisco activity was spectrophotometrically measured according to Walker *et al.* (2013) in 100mM EPPS pH 8.0, 20mM MgCl_2_, 1mM EDTA, 1mM ATP, 5mM creatine phosphate, 20mM NaHCO_3_, 0.5mM ribulose-1,5-bisphosphate, 0.2mM NADH, 12.5U ml^–1^ creatine phosphate kinase, 250U ml^–1^ carbonic anhydrase, 22.5U ml^–1^ phosphoglycerolkinase, 20U ml^–1^ glyceraldehyde-3-phosphodehydrogenase, 56U ml^–1^ triose phosphate isomerase, and 20U ml^–1^ glycerol-3-phosphodehydrogenase.

### Nitrate uptake, content and assimilation

**Fig. 1.**
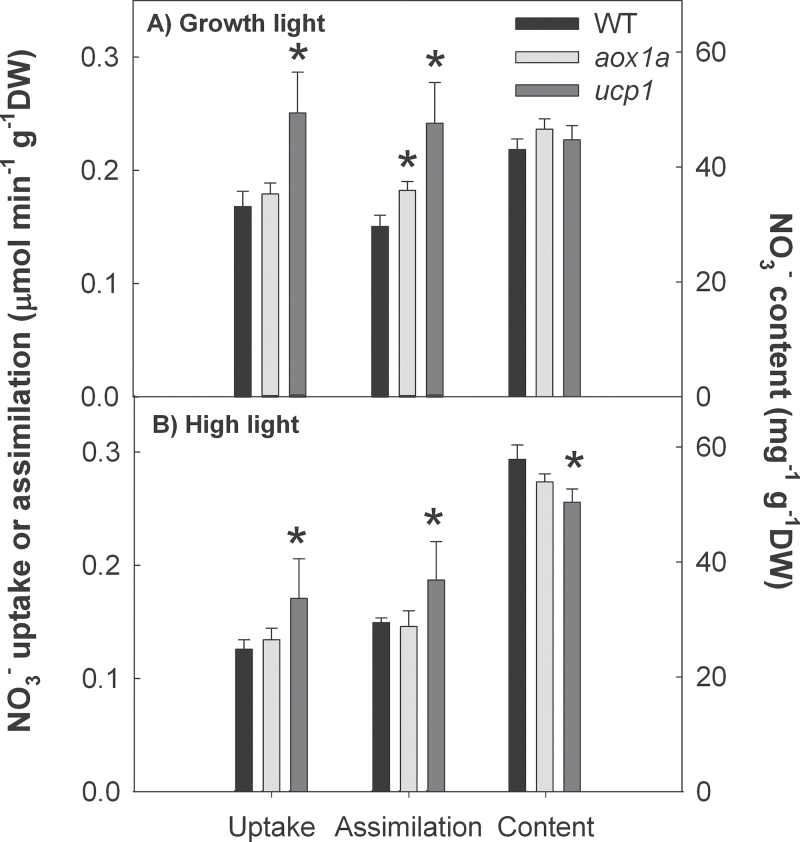
Rates of foliar NO_3_^–^ uptake and assimilation, and free NO_3_^–^ content in wild-type, *aox1a* and *ucp1* shoots of *A. thaliana* fed with ^15^NO_3_^–^ for 6h. Plants were exposed to either growth (A, 160 μmol quanta m^–2^ s^–1^) or saturating (B, 1000 μmol quanta m^–2^ s^–1^) light conditions during the feeding. Shown are the means ±SE of measurements made on five plants. An asterisk denotes a significant difference (*P*<0.05) between genotypes.

WT, *aox1a* and *ucp1 A. thaliana* plants were grown as described above in a hydroponic solution containing 0.2mM NO_3_^–^ at natural abundance ^15/14^N. Subsequently, plants were grown for 24h on a nutrient solution depleted of N (as above). Half of the plants were shifted to a nutrient solution supplemented with 0.2mM NO_3_^–^ enriched 25% with ^15^NO_3_^–^ and the other half were fed natural abundance ^15^NO_3_ as a control. In the first set of experiments, plants were labelled for up to 9h to look at the time course of NO_3_ uptake and assimilation (time effect, Supplementary Fig. S1 available at *JXB* online). Subsequent experiments were limited to 6h of feeding (irradiance effect, Fig. 1). After labelling, plants were rinsed in ultra-pure water then separated into shoots and roots, oven-dried, and ground to a fine powder in a mortar and pestle. Total ^15^N enrichment was measured using an elemental analyser (ECS 4010, Costech Analytical, Valencia, CA, USA) connected directly to a continuous flow isotope ratio mass spectrometer (Delta PlusXP, Thermofinnigan, Bremen, Germany) (Brenna *et al.*, 1997). Isotopic reference materials are interspersed with samples for isotope ratio calibration and acetanilide was used in a multipoint correction for N%.

Free NO_3_^–^ was extracted from powder plant material, and ^15^N enrichment of free NO_3_^–^ was estimated by converting NO_3_^–^ to N_2_O using the denitrifying *Pseudomonas aureofaciens* (Sigman *et al.*, 2001; Casciotti *et al.*, 2002). The headspace N_2_O was sampled with a two-holed needle mounted on an autosampler (GC-PAL, CTC Analytics, Switzerland) and connected to a GasBench II (ThermoFinnigan) interface. Samples were cleaned of water and volatile organic compounds (VOCs) through a liquid nitrogen/ethanol slush trap (–110 °C), and of CO_2_ and H_2_O through an ascarite/magnesium perchlorate trap. Further removal of VOCs was achieved with a Supelco type F trap following the slush trap. Purified samples were separated through a Poraplot Q GC column (Varian, 25 m×0.32mm ID), run through a final nafion water trap (Permapure LLC, NJ) for trace water removal, and analysed by a continuous flow isotope ratio mass spectrometer (Delta PlusV, Thermofinnigan) (Brenna *et al.*, 1997). NO_3_^–^ assimilation was thus estimated as NO_3_^–^ assimilation=total ^15^N–^15^NO_3_^–^. Finally, total free NO_3_^–^ was quantified from powder using high-pressure liquid chromatography (Thayer and Huffaker, 1980).

### Amino acid analysis

Leaf tissues were ground in 0.25ml of 0.1M HCl using a micropestle, and 400 μM aminobutyric acid was added as internal control. Samples were centrifuged for 20min at 24 000 *g* at 4 °C and the supernatant was collected and the pellets re-extracted with 0.25ml of 0.1M HCl. Supernatants were combined and filtered through a 45 μm polyvinylidene difluoride (PVDF) filter and stored at –80 °C until analysis. Amino acids were derivatized using 4-ﬂuoro-7-nitro-2,1,3-benzoxadiazole (NBD-F) according to Aoyama *et al.* (2004) by incubating 5 μl of the extract with 50mM borate buffer pH 9.5 and 3mM NDB-F reagent at 60 °C for 10min. Reactions were terminated by addition of 333mM tartrate buffer pH 2.0 and derivatized amino acids were separated using an Alliance^®^ HPLC System (2695 Separations Module, Waters, Milford, MA, USA) and ﬂuorometrically detected at 540nm with excitation at 470nm (2475 Multi-Wavelength Fluorescence Detector, Waters).

### Feeding system for gas exchange and chlorophyll fluorescence

Twenty-four hours before the gas exchange measurements, plants were transferred to nutrient solution depleted in N source (neither NH_4_^+^ nor NO_3_^–^) for starvation. Subsequently, plants were individually transferred 12h before measurements to stainless steel cuvettes sealed with Teflon caps. The root cuvettes were fed with a continuous flow of nutrient solution supplemented with either NO_3_^–^ or NH_4_^+^ using a custom-built multiplant feeding system. Nutrient solution was equally distributed between each of the six cuvettes using electronically controlled solenoid valves (ASCO RedHat II 8262, Florham Park, NJ, USA). Each individual stainless steel cuvette was housed within a sealed PVC tube containing temperature-controlled circulating water to maintain the root system at 25 °C.

### Gas exchange and chlorophyll fluorescence

Gas exchange and chlorophyll fluorescence measurements were made on fully expanded leaves using a LI6400 (LICOR Biosciences, Lincoln, NE, USA) leaf chamber (LI6400-40). Gas exchange measurements were made at a pO_2_ of 18.6 kPa, a leaf temperature of 25 °C, a saturating light intensity of 1000 μmol quanta m^−2^ s^−1^ PAR (photosynthetically active radiation), and a CO_2_ partial pressure of 37.2 Pa. Light–response curves were made by decreasing light from 2000 to 1500, 1200, 1000, 800, 500, 200, 100, 40, and 20 μmol quanta m^−2^ s^−1^ PAR. The O_2_ response curves were made by modulating pO_2_ inside the chamber using two mass flow controllers (Aalborg, Orangeburg, NY, USA) to mix N and O_2_ gas proportional to 46.6, 32.6, 18.6, 9.3, and 1.9 kPa pO_2_. The order of pO_2_ during the measurements was randomized. Simultaneously, chlorophyll fluorescence measurements were made using a LI6400-40 pulse-modulated fluorometer and multiphase flash protocol (Loriaux *et al.*, 2013). The quantum yield of photosystem II (ϕ_PSII_) and photochemical quenching (qP) were determined as (*F*_m_’–*F*_s_)/*F*_m_’) and (*F*_m_’–F_s_)/(*F*_m_’–F_o_’), respectively. The rate of linear electron transport through PSII (*J*_f_) was calculated from chlorophyll fluorescence measurements as *J*_f_=ϕ_PSII_×*Abs*×*I*×0.48, where *Abs* is leaf absorption (=0.85), *I* is the incident irradiance, and assuming a relative excitation distribution to PSII of 0.48 (Laisk and Loreto, 1996). Furthermore, the rate of electron transport required to sustain the photosynthetic carbon reduction and photorespiratory cycles (*J*_g_) was calculated from gas exchange measurements as *J*_g_=(*A*_net_+*R*_d_) (4*C*_c_ +8Г*)/(*C*_c_–Г*) where *A*_net_ is net CO_2_ assimilation rate, *R*_d_ is dark-type respiratory rate, *C*_c_ is the chloroplastic CO_2_ partial pressure, and Г* is the CO_2_ compensation point in the absence of dark-type respiration.

## Results

### Leaf characteristics

Measurements of growth, leaf chlorophyll content, and Rubisco activity were made to characterize WT, *aox1a*, and *ucp1 A. thaliana* plants grown in hydroponic conditions. Shoot dry weight was 9 and 10% lower in *ucp1* compared with the WT and *aox1a*, respectively (Table 1). However, leaf number, diameter of the rosette, LMA, and root biomass were similar between genotypes. Additionally, leaf chlorophyll content and Rubisco activity were similar, with an average chlorophyll *a*/*b* ratio of 1.8 and Rubisco activity of 48 μmol m^–2^ s^–1^, suggesting similar photosynthetic capacity in all three genotypes (Table 1).

**Table 1.** Growth characteristics, chlorophyll ratio, and Rubisco activity in wild-type, aox1a, and ucp1 Arabidopsis thaliana

	Root biomass (mg)	Shoot biomass (mg)	No. of leaves	Rosette diameter (cm)	LMA (g m^–2^)	Chl *a*/*b*	Rubisco (μmol m^–2^ s^–1^)
WT	88.6±6.9 a	412.3±21.5 a	21±1 a	12.3±1.2 a	68.7±5.4 a	1.7±0.3 a	49.0±3.7 a
*aox1a*	89.8±5.7 a	415.1±18.1 a	21±1 a	12.6±0.8 a	61.7±3.2 a	1.9±0.4 a	44.4±6.4 a
*ucp1*	76.5±5.0 a	375.1±16.0 b	20±1 a	11.3±0.3 a	67.5±5.9 a	1.8±0.3 a	51.0±4.2 a

Values represent the means ±SE of 10 biological replicates for growth characteristics and five biological replicates for chlorophyll and Rubisco measurements.

ANOVA results are indicated; different letters indicate significant differences between genotypes at *P*<0.05.

### Nitrate uptake, accumulation, and assimilation

To estimate the impact of AOX1a and UCP1 function on *de novo* N uptake, rates of NO_3_^–^ uptake and assimilation were measured by feeding hydroponically grown plants ^15^N-enriched NO_3_^–^. The uptake and accumulation of NO_3_^–^ were quantified from measurements of bulk leaf ^15^N and free ^15^NO_3_^–^, respectively, relative to plants fed natural abundance NO_3_^–^. The assimilation of NO_3_^–^ was estimated from differences between total leaf ^15^N minus ^15^NO_3_^–^ content. Rates of NO_3_^–^ uptake and assimilation were measured in WT plants at three time points after initiating feeding (3, 6, and 9h) to determine the optimum time. Rates of shoot NO_3_^–^ uptake, assimilation, and accumulation were similar after 3, 6, and 9h (Supplementary Fig. S1 at *JXB* online). However, rates of root NO_3_^–^ uptake and assimilation were higher at 3h compared with 6h and 9h.

Therefore, NO_3_^–^ uptake, assimilation, and content were measured in WT, *aox1a*, and *ucp1* plants after 6h of ^15^NO_3_^–^ feeding. Under 160 μmol quanta m^–2^ s^–1^ (growth irradiance), shoot NO_3_^–^ uptake was higher in *ucp1* compared with *aox1a* and WT plants. However, shoot assimilation of NO_3_^–^ was higher in both *aox1a* and *ucp1* compared with the WT, while shoot NO_3_^–^ content was similar between all three genotypes (Fig. 1). Regardless of the genotype, the uptake, assimilation, and content of NO_3_^–^ in the roots were similar (Supplementary Fig. S2 at *JXB* online). After 6h under high light (1000 μmol quanta m^–2^ s^–1^), shoot NO_3_^–^ uptake and assimilation were higher in *ucp1* compared with the WT and *aox1a*; however, NO_3_^–^ content was lower (Fig. 1). Under the high light treatment, the root NO_3_^–^ uptake, assimilation, and content were similar between all three genotypes (Supplementary Fig. S2).

### Amino acid analysis

**Fig. 2.**
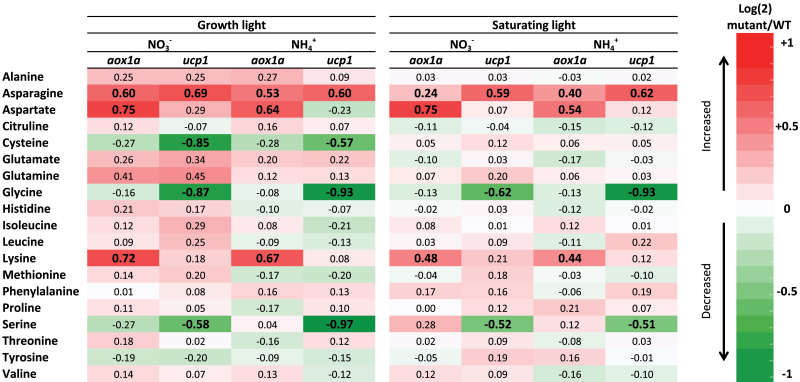
Changes in the content of 19 different amino acids in *aox1a* and *ucp1* leaves of *A. thaliana* fed with either NO_3_^–^ or NH_4_^+^ as sole N source and exposed to either growth (160 μmol quanta m^–2^ s^–1^) or saturating (1000 μmol quanta m^–2^ s^–1^) irradiance for 6h. Values represent the log2 of the mutant to WT ratio. Values in bold indicate differences statistically significantly different between the WT and mutants. (This figure is available in colour at *JXB* online.)

Nineteen amino acids were quantified in leaves of *A. thaliana* WT, *aox1a*, and *ucp1* fed either NO_3_^–^ or NH_4_^+^ as sole N source under both growth (160 μmol quanta m^–2^ s^–1^) and high (1000 μmol quanta m^–2^ s^–1^) irradiance. Asparagine, aspartate, and lysine contents were higher in the *aox1a* mutant plants compared with the WT, regardless of N source or irradiance (Fig. 2; Supplementary Table S1 at *JXB* online). However, only asparagine was significantly higher in *ucp1* compared with the WT plants. In contrast, under growth irradiance, cysteine, glycine, and serine were lower in *ucp1* compared with the WT, regardless of the N source. However, under saturating irradiance, glycine and serine were significantly decreased but cysteine was not in *ucp1* compared with WT plants.

### Response of photosynthesis

**Fig. 3.**
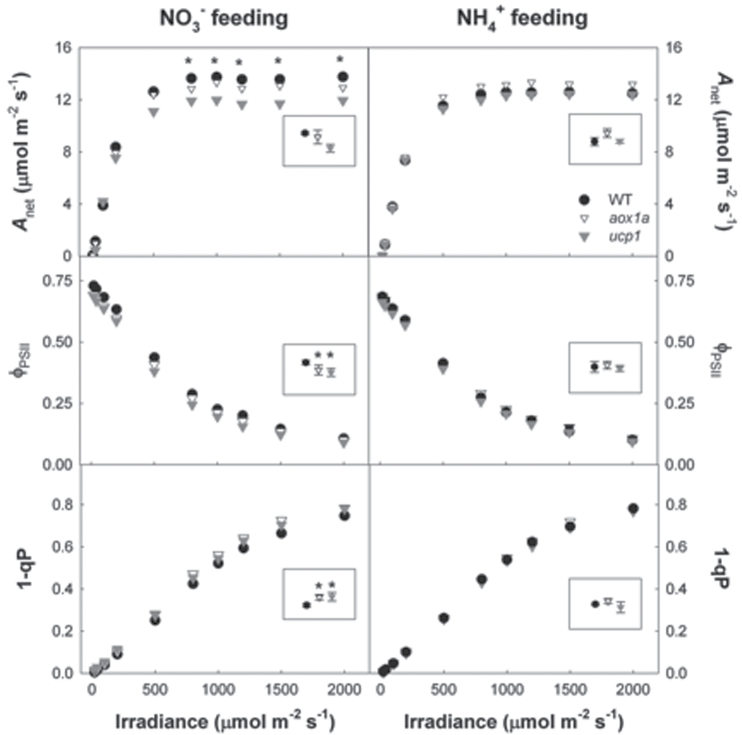
Net CO_2_ assimilation rate (*A*_net_), quantum yield (ϕ_PSII_), and excitation pressure (1–qP) in response to light intensity in wild-type, *aox1a*, and *ucp1* leaves of *A. thaliana* fed either NO_3_^–^ or NH_4_^+^ as sole N source. Shown are the means of measurements made on three plants. The inset box within each panel presents the grand mean with the standard error, estimated from the MSE term in the ANOVA. Significant differences (*P*<0.05) were denoted by an asterisk either in the inset box when the genotype effect was significant or on the graph to indicate a significant genotype×irradiance interaction.

**Fig. 4.**
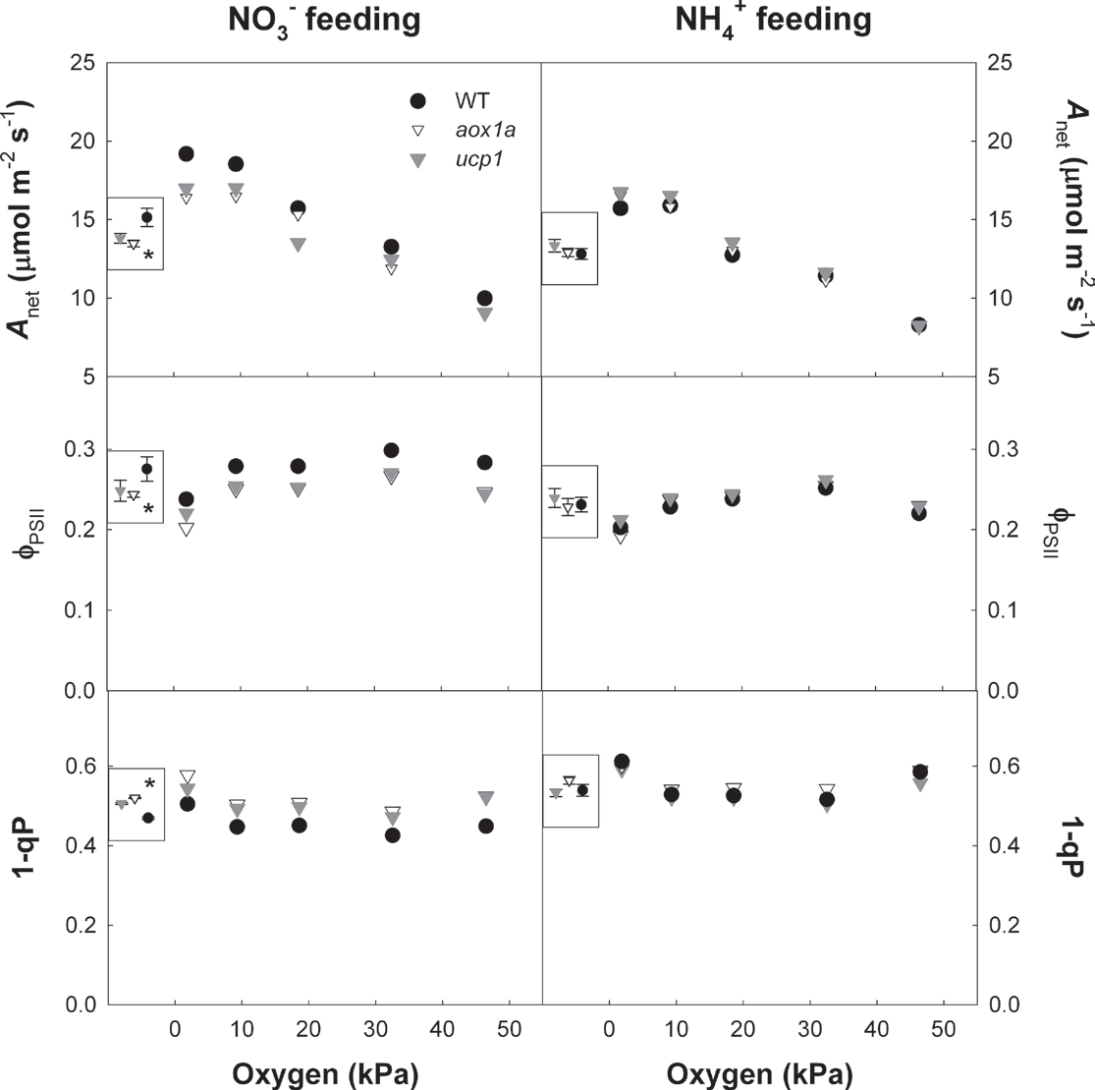
Net CO_2_ assimilation rate (*A*_net_), quantum yield (ϕ_PSII_), and excitation pressure (1–qP) in response to O_2_ atmospheric partial pressure in wild-type, *aox1a*, and *ucp1* leaves of *A. thaliana* fed with either NO_3_^–^ or NH_4_^+^ as sole N source. Shown are the means ±SE of measurements made on three plants. The inset box within each panel presents the grand mean with the standard error, estimated from the MSE term in the ANOVA. An asterisk denotes a significant difference (*P*<0.05) between genotypes.

Measurements of *A*_net_, ϕ_PSII_, and the excitation pressure on the chloroplast electron transport chain (1–qP) were measured in WT, *aox1a*, and *ucp1* plants fed either NO_3_^–^ or NH_4_^+^ to test the impact of two major electron sinks on photosynthesis. In WT plants, *A*_net_ and ϕ_PSII_ increased while 1–qP decreased under NO_3_^–^ compared with NH_4_^+^ feeding (Fig. 3). However, in the *aox1a* and *ucp1* plants the change in N source had no effect on *A*_net_, ϕ_PSII_, or 1–qP. Under NO_3_^–^ and saturating irradiance, *A*_net_ was lower in *ucp1* compared with WT and *aox1a* plants; however, under non-saturating irradiances, *A*_net_ was similar between genotypes. Furthermore, under NO_3_^–^, the measured ϕ_PSII_ was lower and 1–qP higher in the *ucp1* and *aox1a* plants compared with the WT plants, regardless of irradiance. Under NH_4_^+^ feeding, *A*_net_, ϕ_PSII_, and 1–qP were not significantly different between *ucp1*, *aox1a*, and WT plants across all irradiances. There was a significant oxygen response of *A*_net_ but not ϕ_PSII_ and 1–qP for all three genotypes regardless of N form (Fig. 4). In WT plants *A*_net_ and ϕ_PSII_ were higher while 1–qP was lower under NO_3_^–^ compared with NH_4_^+^ feeding across all pO_2_; however, there was no difference in these parameters between N form in the *ucp1* and *aox1a* plants (Fig. 4). Additionally, there was a significant difference in *A*_net_, ϕ_PSII_, and 1–qP between WT and both mutant lines (*ucp1* and *aox1a*) at all pO_2_ under NO_3_^–^ but not NH_4_^+^.

**Fig. 5.**
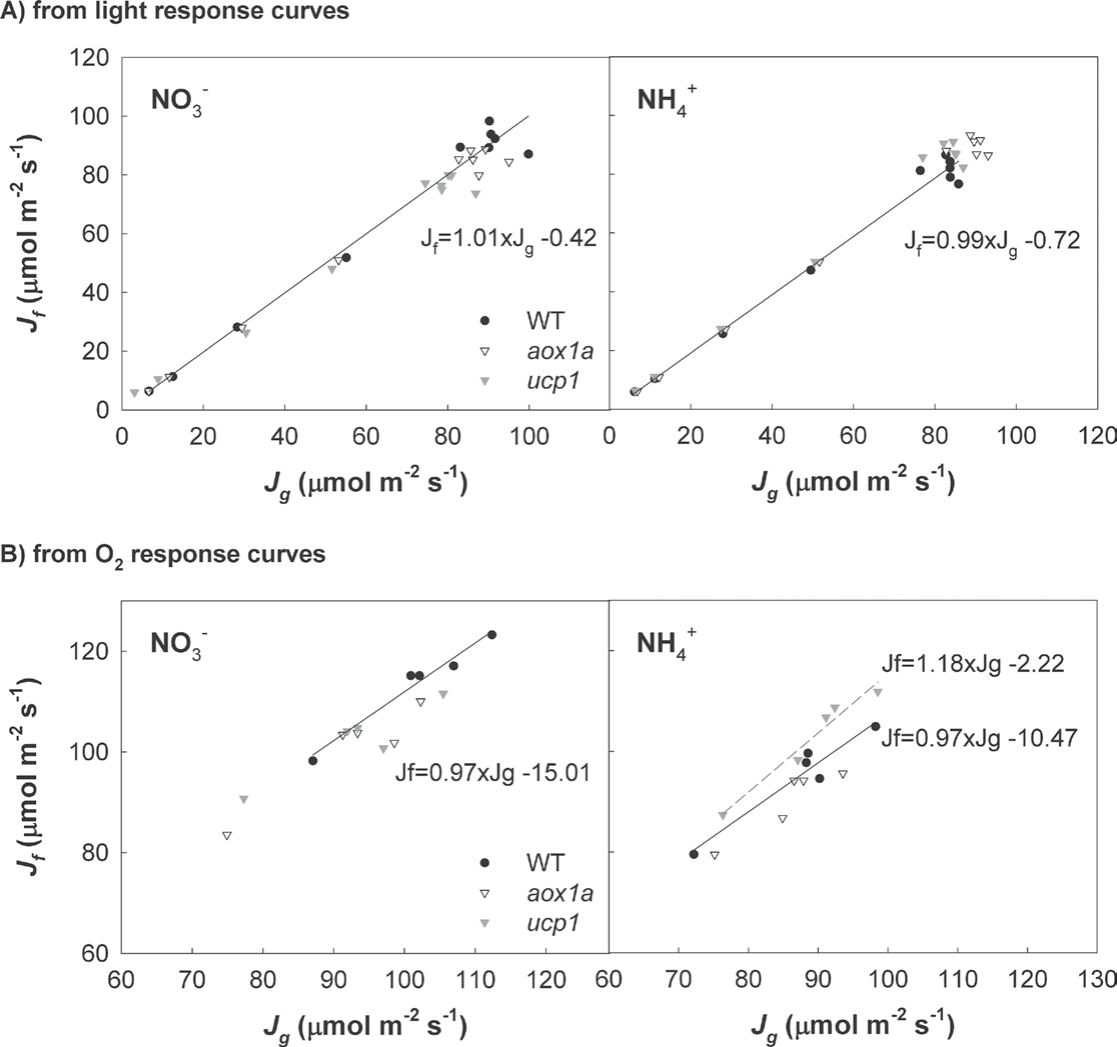
Correlation between the electron transport rate through PSII (*J*_f_) and the electron transport rate required for CO_2_ assimilation and photorespiration (*J*_g_) in wild-type, *aox1a*, and *ucp1* leaves of *A. thaliana* fed with either NO_3_^–^ or NH_4_^+^ as sole N source. Correlation was established in response to light intensity (A; from 2000 to 20 μmol quanta m^–2^ s^–1^) and O_2_ partial pressure (B; from 46.6 to 1.9 kPa pO_2_).

The rate of linear electron transport through PSII estimated from chlorophyll fluorescence (*J*_f_) was compared with the electron transport demand required to sustain rates of CO_2_ assimilation and photorespiration (*J*_g_). The relationship between *J*_f_ and *J*_g_ was linear in response to irradiance and did not differ between NO_3_^–^ and NH_4_^+^ feeding for all three genotypes (Fig. 5A). Additionally, the relationship between *J*_f_ and *J*_g_ under NO_3_^–^ feeding was similar between all three genotypes in response to pO_2_; however, under NH_4_^+^ feeding, there was a significantly higher slope in the *ucp1* plants (1.18) compared with *aox1a* (0.95) and WT (0.97) plants (Fig. 5B).

## Discussion

The disruption of mAET had significant impacts on both foliar N and carbon metabolism in *A. thaliana*. For example, the loss of the uncoupling protein UCP1 or the alternative oxidase AOX1a increased rates of foliar NO_3_^–^ assimilation compared with WT plants. Additionally, *A*_net_ was lower in *ucp1* plants compared with the WT and *aox1a* lines in NO_3_^–^-fed plants. However, under NH_4_^+^, rates of *A*_net_ were not significantly different between genotypes. As discussed below, these data demonstrate that UCP1 and AOX1a are important for balancing the energy partitioning between N and carbon metabolism.

### Disruption of alternative mitochondrial electron transport enhances foliar nitrate assimilation

The rates of foliar NO_3_^–^ assimilation were higher in the *aox1a* and *ucp1* plants compared with the WT under the non-saturating growth light (160 μmol photons m^–2^ s^–1^) conditions (Fig. 1). Low light conditions would probably decrease the amount of reductant exported from the chloroplast, and the competition for reductant between NO_3_^–^ assimilation and mitochondrial electron transport would be high. Under these conditions, the availability of NADH for the cytosolic conversion of NO_3_^–^ to NO_2_^–^ and the chloroplastic reduction of NO_2_^–^ to NH_4_^+^ by ferredoxin would be limiting. Therefore, the increase in NO_3_^–^ assimilation in the *upc1* and *aox1a* plants under low light is probably attributed to an increased availability of cytosolic reductant because of decreased consumption by the mitochondria. The increased NO_3_^–^ assimilation in the *ucp1* and *aox1a* plants also corresponded to a higher amino acid content (Fig. 2, Supplementary Table S1 at *JXB* online) and lower total leaf carbon to N ratio as previously observed in tobacco plants deficient in the mitochondrial complex I (CMS mutant) (Dutilleul *et al.*, 2005) and in AOX1a (Watanabe *et al.*, 2008). Alternatively, at saturating light (1000 μmol photons m^–2^ s^–1^), the export of excess reductant from the chloroplast into the cytosol will be high and will not limit rates for NO_3_^–^ assimilation. In fact under high light, there was only a small increase in NO_3_^–^ assimilation with the loss of UCP1 and there was no change in NO_3_^–^ assimilation with the absence of AOX1a.

Generally, the effect of lack of UCP1 on N metabolism was more marked than that of the lack of AOX1a. The expression of *UCP1* has been shown to be up-regulated in the *aox1a* mutant (Watanabee *et al.*, 2008, 2010). Given the potentially overlapping function of these two respiratory components, UCP1 may partly compensate for the loss of AOX1a, which would therefore diminish the impact of the genetic manipulation. However, AOX content has been shown to decrease in the *ucp1* mutant (Sweetlove *et al.*, 2006). The higher rates of foliar NO_3_^–^ assimilation in the *ucp1* and *aox1a* plants also require an increase in carbon skeletons for the *de novo* synthesis of amino acids. The present results show a significant increase in aspartate and asparagine levels in mutants compared with the WT. The aspartate pathway drives the synthesis of several amino acids that may contribute to generate additional energy under stress conditions (Galili, 2011). This pathway has been suggested to operate in combination with the TCA cycle in inducing the catapleurotic fluxes with energy deprivation conditions. However, anapleurotic fluxes can also supply amino acid synthesis with carbon skeletons. In *aox1a* and *ucp1* mutants, it is likely that asparagine family synthesis was driven by reductant accumulation and carbon skeleton availability leaking out of the TCA cycle. It has been demonstrated that the carbon needed for the synthesis of amino acids comes primarily from the partial operation of the TCA cycle (Chen and Gadal, 1990; Tcherkez *et al.*, 2009; Sweetlove *et al.*, 2010). Additionally, it has been reported that there is an increase in the anaplerotic production of carbon skeletons through an incomplete TCA cycle with increased NO_3_^–^ assimilation (Scheible *et al.*, 1997; Stitt, 1999). Therefore, the increase in NO_3_^–^ assimilation and overall amino acid content observed in the *ucp1* and *aox1a* lines probably increased the TCA production of carbon skeletons. This would further increase NADH production available via the malate shuttle for *de novo* NO_3_^–^ assimilation.

Root NO_3_^–^ assimilation was not altered by the loss of UCP1 and AOX1a, suggesting a more important role for AOX1a and UPC1 in photosynthetic tissue. This is supported by 6-fold higher AOX1a transcripts in the shoot compared with the root in *A. thaliana*; however, the UCP1 expression level is similar between root and shoot tissues (Watanabe *et al.*, 2010). Furthermore, the AOX1d transcript level is enhanced in the root of *aox1a* mutants, but not in the shoot, suggesting a potential compensatory effect for the loss of AOX1a in the roots (Watanabe *et al.*, 2010). Taken together, AOX1a and potentially UCP1 appears to have a more predominant role in shoot than root tissues and plays an important role in regulating *de novo* shoot assimilation of NO_3_^–^, particularly when the reductant availability is limiting.

### Alternative mitochondrial electron transport and nitrate assimilation synergistically optimize photosynthesis

The present results demonstrate that both NO_3_^–^ assimilation and mAET optimize rates of photosynthetic CO_2_ assimilation; however, independently neither is sufficient to influence *A*_net_. For example, *A*_net_ was higher under NO_3_^–^ compared with NH_4_^+^ feeding in WT plants but not in the *aox1a* and *ucp1* mutants, despite increased NO_3_^–^ assimilation in these plants. Additionally, under NH_4_^+^ feeding, there was not a significant difference in *A*_net_ between the WT and the two mutant lines (*ucp1* and *aox1a*). This suggests that the increase in *A*_net_ under NO_3_^–^ is dependent on functional AOX1A and UCP1, and that NO_3_^–^ assimilation alone is insufficient to alter *A*_net_. Furthermore, the loss of AOX1A and UCP1 had no effect on *A*_net_ or leaf photochemistry under NH_4_^+^.

In WT plants, *A*_net_ and ϕ_PSII_ were 12–17% higher under NO_3_^–^- compared with NH_4_^+^-fed plants, respectively. An increase in *A*_net_ and ϕ_PSII_ under NO_3_^–^ versus NH_4_^+^ feeding has been previously described in barley (Bloom *et al.*, 1989), wheat (Bloom *et al.*, 2002), tomato (Searles and Bloom, 2003), and maize (Cousins and Bloom, 2003). The foliar reduction of NO_3_^–^ to NH_4_^+^ requires cytosolic NADH, typically generated from reductant exported from the chloroplast, and ferredoxin within the chloroplast. Therefore, NO_3_^–^ assimilation may compete for reductant with Rubisco-mediated assimilation of CO_2_ (Bloom *et al.*, 2002). However, under the conditions used here and as previously reported (Bloom *et al*., 1989, 2002; Cousins and Bloom, 2003; Searles and Bloom, 2003), the rates of *A*_net_ were higher in NO_3_^–^- versus NH_4_^+^-fed plants. This suggests that the consumption of reductant via NO_3_^–^ assimilation stimulates rates of net CO_2_ assimilation and ϕ_PSII_, probably through optimizing the ATP/NADPH production within chloroplasts. Additionally, the present data indicate that NO_3_^–^ assimilation contributes to protect the chloroplast electron transport chain from over-reduction and therefore ensures optimal rates of CO_2_ assimilation.

The measured *A*_net_ was lower in NO_3_^–^-fed *ucp1* mutants compared with WT plants; however, there was no difference in *A*_net_ between *ucp1* and WT plants under NH_4_^+^ feeding (Fig. 3). The decrease in *A*_net_ seen under NO_3_^–^ feeding is consistent with these plants grown in soil as reported by Sweetlove *et al.* (2006). These authors attributed the decrease in *A*_net_ in the *ucp1* compared with the WT to a restricted flux through the photorespiratory pathway and an associated limited regeneration of ribulose-1,5-bisphosphate. A decrease in the glycine to serine conversion could affect methionine synthesis through C1 metabolism. Both serine and methionine are precursors of cysteine synthesis, which is at a low level in *ucp1* compared with the WT. These data are similar to those of Sweetlove *et al.* (2006), who also showed a lower amount of glycine and serine in the *ucp1* mutant compared with the WT (Fig. 2). However, it was found that the difference in *A*_net_ between the *ucp1* mutant and the WT was constant in response to O_2_ availability (from 1.9 kPa to 46.6 kPa O_2_). This suggests that UCP1 optimizes *A*_net_ regardless of rates of photorespiration and probably plays an important role in balancing the energy supply with demand between N and carbon metabolism within the leaf. The N feeding experiments demonstrated that NO_3_^–^ assimilation could compensate for the lack of excess reductant consumption by the mitochondria in the absence of UCP1. However, *A*_net_ was not enhanced in *aox1a* and *ucp1* mutants fed with NO_3_^–^ as seen in WT plants, suggesting that the stimulation of CO_2_ fixation by NO_3_^–^ assimilation is dependent on a fully functional mAET.

In the *ucp1* mutants fed with NH_4_^+^, the electron production by the chloroplastic electron transport chain increased significantly with decreasing oxygen compared with the electron demand for *A*_net_ and photorespiration (Fig. 5B). This shift between electron production and demand indicates extra electron transport to alternative chloroplastic sinks such as the Mehler reaction. In addition to the Mehler reaction, other alternative electron sinks such as the cyclic electron flux and chlororespiration have been reported to optimize ATP synthesis, balance the production of the chloroplastic ratio of ATP/NADPH, and avoid over-reduction of the photosynthetic electron transport chain (Johnson, 2005). In *ucp1* mutants fed NH_4_^+^ under non-photorespiratory conditions, the change in *J*_f_/*J*_g_ suggests that the Mehler reaction has a greater influence on linear electron flow compared with other genotypes and treatments. This would avoid excess NADPH accumulation within the stroma due to the loss of two other major electron sinks (mitochondria alternative pathways and NO_3_^–^ assimilation) while still maintaining rates of linear electron transport.

## Conclusion

In summary the importance of mAET in foliar *de novo* NO_3_^–^ assimilation in *A. thaliana*, particularly under conditions that limit reductant availability (low light), is demonstrated in the present work. Additionally, the data show that both mAET and NO_3_^–^ assimilation influence rates of photosynthetic CO_2_ assimilation and electron transport. mAET and NO_3_^–^ assimilation appear to function synergistically to avoid excess reductant accumulation and over-reduction of the chloroplast. Finally, the data demonstrate that mitochondrial respiration significantly contributes to the energy balancing between N and carbon metabolism.

## Supplementary data

Supplementary data are available a*t JXB* online.

Figure S1. Rates of NO_3_^–^ uptake and assimilation, and free NO_3_^–^ content in wild-type shoots and roots of *A. thaliana* fed with ^15^NO_3_^–^ for 3, 6, or 9h.

Table S1. Amino acid levels (nmol mg^–1^ DW) in shoots of wild-type, *aox1a*, and *ucp1 Arabidopsis thaliana* fed either NO_3_^–^ or NH_4_^+^ as sole N source.

Figure S2. Rates of NO_3_^–^ uptake and assimilation, and free NO_3_^–^ content in wild-type, *aox1a*, and *ucp1* roots of *A. thaliana* fed with ^15^NO_3_^–^ for 6h.

Supplementary Data

## Abbreviations:

*A*_net_: net CO_2_ assimilation
AOX: alternative oxidase
*J*_f_: rate of linear electron transport through PSII calculated from chlorophyll fluorescence
*J*_g_: rate of electron transport required to sustain the photosynthetic carbon reduction and photorespiration
LMA: leaf mass per area
mAET: mitochondrial alternative electron transport
N: nitrogen
qP: photochemical quenching
UCP: uncoupling protein
ϕPSII: quantum yield of photosystem II
1–qP: excitation pressure on PSII
