# Supplementary material for: Disruption of the mitochondrial alternative oxidase (AOX) and uncoupling protein (UCP) alters rates of foliar nitrate and carbon assimilation in Arabidopsis thaliana
Source: J Exp Bot. 2014 May 5;65(12):3133–42. doi: 10.1093/jxb/eru158 (PMC4071831; doi:10.1093/jxb/eru158)
Supplement: Supplementary Data [file supp_65_12_3133__index.html]

Disruption of the mitochondrial alternative oxidase (AOX) and uncoupling protein (UCP) alters rates of foliar nitrate and carbon assimilation in Arabidopsis thaliana — Disruption of the mitochondrial alternative oxidase (AOX) and uncoupling protein (UCP) alters rates of foliar nitrate and carbon assimilation in Arabidopsis thaliana — Supplementary Data 

# Disruption of the mitochondrial alternative oxidase (AOX) and uncoupling protein (UCP) alters rates of foliar nitrate and carbon assimilation in *Arabidopsis thaliana*

## Supplementary Data

Data files

**Files in this Data Supplement:**

- Supplementary Data - Supplementary Data
