# Supplementary material for: Disruption of the mitochondrial alternative oxidase (AOX) and uncoupling protein (UCP) alters rates of foliar nitrate and carbon assimilation in Arabidopsis thaliana
Source: J Exp Bot. 2014 May 5;65(12):3133–42. doi: 10.1093/jxb/eru158 (PMC4071831; doi:10.1093/jxb/eru158)
Supplement: Supplementary Data [file supp_eru158_jexbot120873_file001.pdf]

Disruption of the mitochondrial alternative oxidase (AOX) and uncoupling protein (UCP) alter rates of foliar nitrate and carbon assimilation in *Arabidopsis thaliana*. *Anthony Gandin, Mykhaylo Denysyuk, and Asaph Cousins*

SUPPLEMENTARY DATA

Supplemental table S1. Amino acid levels (nmol mg^{-1}DW) in shoots of wild type, *aox1a* and *ucp1 Arabidopsis thaliana* fed either NO_3^- or NH_4^+ as sole N source. During the feeding experiments plants were exposed to either growth (160 µmol quanta m^{-2} s^{-1}) or saturating (1000 µmol quanta m^{-2} s^{-1}) irradiance. Values are presented as mean ± SE.

	Growth light											
	NO_3^-						NH_4^+					
	WT		*aox1a*		*ucp1*		WT		*aox1a*		*ucp1*	
**Alanine**	4.2	± 0.6	5.0	± 0.5	5.0	± 0.5	3.8	± 0.5	4.6	± 0.5	4.1	± 0.6
**Asparagine**	18.2	± 2.3	27.6	± 2.2	29.3	± 2.1	19.8	± 2.0	28.5	± 2.3	30.0	± 1.8
**Aspartate**	7.6	± 1.1	12.7	± 1.2	9.3	± 1.2	6.1	± 1.1	9.5	± 1.2	5.2	± 1.1
**Citruline**	0.3	± 0.1	0.3	± 0.1	0.3	± 0.1	0.2	± 0.1	0.2	± 0.1	0.2	± 0.1
**Cysteine**	0.3	± 0.1	0.3	± 0.0	0.2	± 0.1	0.2	± 0.1	0.2	± 0.1	0.1	± 0.0
**Glutamate**	19.3	± 2.4	23.0	± 2.0	24.4	± 2.3	17.6	± 2.4	20.3	± 1.9	20.6	± 2.5
**Glutamine**	4.9	± 0.2	6.5	± 0.1	6.6	± 0.1	4.6	± 0.2	5.0	± 0.1	5.1	± 0.2
**Glycine**	0.6	± 0.1	0.5	± 0.1	0.3	± 0.1	0.6	± 0.1	0.5	± 0.1	0.3	± 0.1
**Histidine**	1.7	± 0.4	2.0	± 0.4	1.9	± 0.4	1.0	± 0.4	0.9	± 0.4	0.9	± 0.5
**Isoleucine**	3.0	± 1.2	3.3	± 1.3	3.7	± 1.4	5.2	± 1.4	5.5	± 1.2	4.5	± 1.2
**Leucine**	1.7	± 0.2	1.8	± 0.2	2.0	± 0.2	1.7	± 0.2	1.6	± 0.2	1.5	± 0.2
**Lysine**	2.3	± 0.4	3.8	± 0.4	2.6	± 0.4	1.3	± 0.4	2.1	± 0.4	1.4	± 0.5
**Methionine**	2.8	± 0.6	3.1	± 0.6	3.2	± 0.6	2.8	± 0.7	2.5	± 0.6	2.4	± 0.6
**Phenylalanine**	5.0	± 1.0	5.0	± 0.9	5.3	± 1.1	3.2	± 1.1	3.5	± 1.2	3.5	± 0.9
**Proline**	1.8	± 0.1	1.9	± 0.1	1.9	± 0.1	2.0	± 0.1	1.7	± 0.1	2.1	± 0.1
**Serine**	2.6	± 0.4	2.2	± 0.5	1.8	± 0.5	3.4	± 0.4	3.5	± 0.4	1.7	± 0.4
**Threonine**	2.5	± 0.7	2.8	± 0.6	2.5	± 0.6	2.4	± 0.7	2.1	± 0.6	2.6	± 0.7
**Tyrosine**	4.5	± 0.5	4.0	± 0.6	3.9	± 0.5	5.1	± 0.5	4.8	± 0.6	4.6	± 0.6
**Valine**	4.2	± 0.6	4.6	± 0.6	4.4	± 0.6	5.1	± 0.5	5.5	± 0.5	4.7	± 0.6

Saturating light												
	NO_3^-						NH_4^+					
	WT		*aox1a*		*ucp1*		WT		*aox1a*		*ucp1*	
**Alanine**	4.2	± 0.5	4.3	± 0.5	4.3	± 0.6	4.2	± 0.5	4.1	± 0.5	4.2	± 0.6
**Asparagine**	17.9	± 1.8	21.2	± 1.9	26.9	± 1.9	16.9	± 2.3	22.4	± 2.2	26.0	± 2.4
**Aspartate**	7.5	± 1.2	12.6	± 1.1	7.8	± 1.3	6.9	± 1.1	10.0	± 1.2	7.5	± 1.3
**Citruline**	0.1	± 0.1	0.1	± 0.1	0.1	± 0.1	0.1	± 0.1	0.1	± 0.1	0.1	± 0.1
**Cysteine**	0.2	± 0.6	0.2	± 0.1	0.2	± 0.0	0.3	± 0.1	0.3	± 0.1	0.3	± 0.1
**Glutamate**	20.8	± 2.1	19.4	± 2.4	21.2	± 2.4	19.2	± 2.0	17.0	± 2.3	18.7	± 2.3
**Glutamine**	4.6	± 0.2	4.8	± 0.2	5.2	± 0.2	4.7	± 0.1	4.9	± 0.2	4.8	± 0.2
**Glycine**	0.6	± 0.1	0.5	± 0.1	0.4	± 0.1	0.5	± 0.1	0.5	± 0.1	0.3	± 0.1
**Histidine**	1.5	± 0.4	1.5	± 0.5	1.6	± 0.4	1.7	± 0.4	1.6	± 0.4	1.7	± 0.5
**Isoleucine**	2.1	± 1.3	2.2	± 1.2	2.1	± 1.2	3.2	± 1.4	3.5	± 1.2	3.2	± 1.2
**Leucine**	1.4	± 0.2	1.4	± 0.2	1.5	± 0.2	1.7	± 0.2	1.6	± 0.2	1.9	± 0.2
**Lysine**	2.6	± 0.5	3.6	± 0.4	3.0	± 0.5	2.6	± 0.5	3.6	± 0.5	2.9	± 0.5
**Methionine**	1.7	± 0.6	1.7	± 0.7	1.9	± 0.6	2.8	± 0.7	2.7	± 0.7	2.6	± 0.7
**Phenylalanine**	5.1	± 1.1	5.8	± 1.1	5.7	± 1.0	4.5	± 1.0	4.3	± 1.2	5.1	± 1.0
**Proline**	2.0	± 0.1	2.0	± 0.1	2.2	± 0.1	2.0	± 0.1	2.3	± 0.1	2.1	± 0.1
**Serine**	2.4	± 0.5	2.9	± 0.4	1.6	± 0.4	2.4	± 0.4	2.6	± 0.5	1.7	± 0.4
**Threonine**	3.5	± 0.7	3.5	± 0.8	3.7	± 0.7	2.3	± 0.6	2.1	± 0.6	2.3	± 0.7
**Tyrosine**	3.9	± 0.5	3.7	± 0.6	4.4	± 0.6	4.7	± 0.7	5.3	± 0.6	4.7	± 0.5
**Valine**	4.3	± 0.6	4.7	± 0.6	4.6	± 0.6	5.1	± 0.5	4.5	± 0.5	4.7	± 0.6

Figure S1

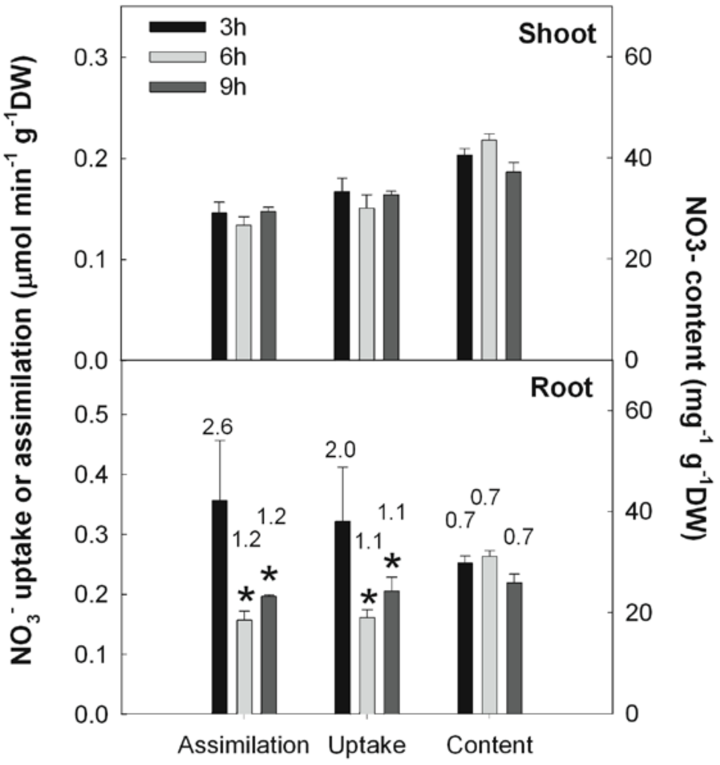

Figure S1. Rates of NO_3^- uptake and assimilation, and free NO_3^- content in wild-type shoots and roots of *A. thaliana* fed with $^{15}NO_3^-$ for 3, 6 or 9 hrs. Numbers represent the root to shoot ratios. Shown are the means ± the SE of measurements made on five plants. An asterisk denotes a significant difference for P<0.05.

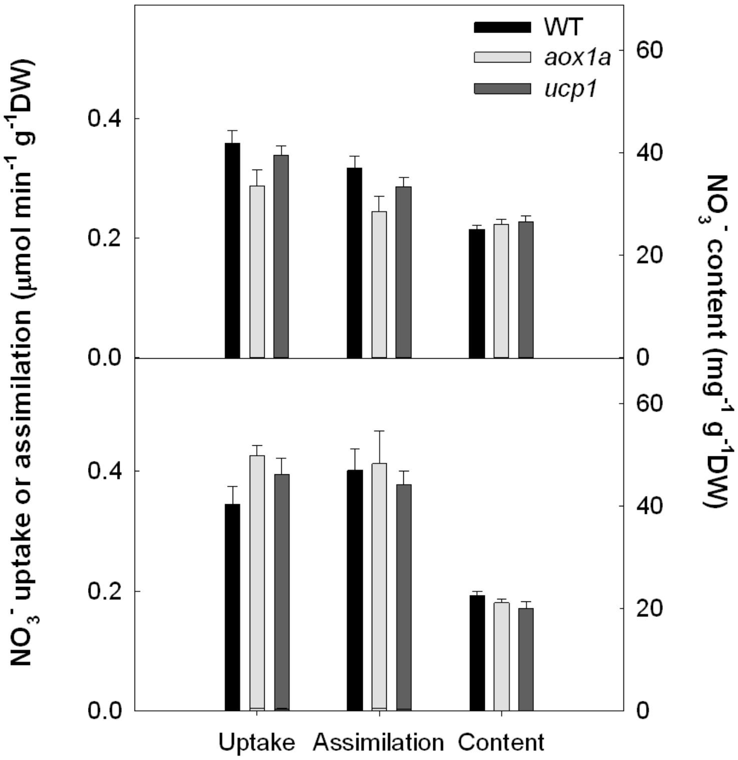

Figure S2. Rates of NO_3^- uptake and assimilation, and free NO_3^- content in wild-type, *aox1a* and *ucp1* roots of *A. thaliana* fed with $^{15}NO_3^-$ for 6 hrs. Plants were exposed to either growth (A, 160 µmol quanta m^{-2} s^{-1}) or saturating (B, 1000 µmol quanta m^{-2} s^{-1}) light conditions during the feeding. Shown are the means ± the SE of measurements made on five plants. An asterisk denotes a significant difference for P<0.05.
